# The Preparation of Modified Industrial Waste Polyacrylonitrile for the Adsorptive Recovery of Pt(IV) from Acidic Solutions

**DOI:** 10.3390/ma9120988

**Published:** 2016-12-06

**Authors:** Sung Il Yoon, Sok Kim, Chul-Woong Cho, Yeoung-Sang Yun

**Affiliations:** 1 Division of Semiconductor and Chemical Engineering, Chonbuk National University, Jeonbuk 54896, Korea; ironsavior@jbnu.ac.kr (S.I.Y.); sokkim81@korea.ac.kr (S.K.); 2 Division of Environmental Science and Ecological Engineering, College of Life Science and Biotechnology, Korea University, Seoul 02841, Korea

**Keywords:** waste textile, recovery, Pt(IV), surface modification

## Abstract

Sorption technique is one of the most effective methods for recovering precious metals from wastewater solutions; however, its main drawbacks of the traditional sorbents are the slow kinetics and relatively low sorption capacities. As a solution, thin sorbent fibers have been highlighted because they can lead to fast adsorption kinetics due to their high surface areas and numerous binding sites. In this sense, the applicability of an industrial waste polyacrylonitrile (PAN) textile was examined to recover Pt(IV) from acid solutions. In order to enrich cationic functional groups on the surface of a PAN textile, the textile was chemically modified via polyethylenmine (PEI) coating. Afterwards, using PEI-coated PAN fiber, batch sorption experiments (isotherms and kinetics) and column experiments were conducted to evaluate its sorption performance toward Pt(IV). It was clearly revealed in column experiments that the PEI-coated waste PAN textile (WPAN) has fast kinetics and good performance for Pt(IV) recovery.

## 1. Introduction

Typical methods of removing ionic solutes such as heavy metals, precious metals, and dye from aqueous solutions include precipitation, ion-exchange, filtration, and sorbent extraction [1,2]. Among them, the adsorption approach is promising because it is very effective, while it may not generate secondary pollution. In the adsorption process, the selection or development of sorbents is most important because removal/recovery efficiency and cost-effectiveness are determined mainly by the performance of adsorbents. Therefore, researchers have been developing several sorbents considering environmental friendliness, cost-effectiveness, and high removal efficiency. Some of them have succeeded in developing environmentally benign and high-performance sorbents using fermentation waste yeasts [3]; however, these sorbents are in powder form and difficult to apply in the sorption process, especially in the packed column type process. Thus, the powder-form sorbents usually face problems such as pressure drops and clogging in column experiments. For these reasons, the sorbents need to be immobilized [2] by forming them as capsules, beads, or fibers [4,5,6]. In general, the immobilization process mostly uses organic solvents or reactive chemicals, rendering the overall process cost-ineffective and environmentally unfriendly, though the resulting sorbents do have good sorption capability. Hence, a simpler and more cost-effective approach that has greener immobilization processes or uses spent waste fibers is needed for sorbent development. 

Among the various adsorbent shapes, adsorbent fibers can lead to very fast adsorption kinetics and abilities for binding some micropollutants because they have a large surface area and many binding sites. Moreover, a higher proportion of the sorbent’s sorption capacity can be effectively used in the column process and can quickly remove or recover target materials. Another advantage of the material is that the resource from which the adsorbent fiber is manufactured is abundant in the world. Indeed, textile waste has been generated in large amounts of >64 million tons per year; however, they have been recycled in ineffective ways, e.g., depolymerization (for use of polymer raw materials) and incineration (for use as an energy source) [7]. 

Therefore, in light of fiber-type adsorbent development and resource recycling, waste textiles have been applied to develop adsorbents with numerous positively charged functional groups. Here, to enrich the positive groups on the surface of a waste polyacrylonitrile (PAN) fiber, polyethyleneimine (PEI) was coated on a waste textile, and adsorption capacities of the designed sorbent were then tested using a negatively charged solute in batch and column experiments. As a model for negatively charged solutes, Pt(IV) was selected because it is one of the most typical precious metals, widely used as a catalyst in several industrial fields, e.g., organic synthesis, electrical industries, electronic devices, medical prostheses, and the automotive industry [8]. 

## 2. Materials and Methods

### 2.1. Materials

A waste PAN textile (WPAN) was obtained from Wet-Processing Laboratory directed by Prof. Yong Sik Chung at the Chonbuk National University, Jeonju, Korea. Polyethylenimine (PEI) (M.W.: 70,000, branch, content: 50%) was purchased from Hapjeong Moolasn Co., Ltd. (Seoul, Korea). Glutaraldehyde (GA) (content: 25%) was purchased from Junsei Chemical Co., Ltd. (Tokyo, Japan). Potassium (IV) hexachloroplatinate (K_2_PtCl_6_) (content: 99.0%) was purchased from Kojima Chemicals Co., Ltd. (Sayama, Japan). Sodium hydroxide (content: 97%) and hydrochloric acid (content: 35%) was purchased from Daejung Chemicals & Metals Co., Ltd. (Siheung, Korea). Lewatit MonoPlus M500 (M500) was supplied by Samsung BP Chemicals Co., Ltd. (Ulsan, Korea), and its speciation is given in Table 1.

### 2.2. Surface Modification of Waste Textile

Hydrolyzed waste PAN textile (HWPAN) was prepared by reacting 2 g (dry weight) of the waste PAN textile with 100 mL of a 5 M NaOH solution for 35–180 min at 80 °C and 200 rpm. After the reaction, the HWPAN was washed with 1 L of deionized water. A PEI-coated waste PAN textile (PWPAN) was then prepared by coating HWPAN (0.2 g) with 200 mL of PEI solution (13 g/L) under constant shaking for 4 h at 40 °C and 160 rpm, and it was subsequently cross-linked with 0.5 mL of a GA solution under constant shaking for 3 h at 40 °C and 160 rpm. The solution pH was maintained at 10.5 throughout the reaction. The prepared PWPAN was washed with 1 L of deionized water and freeze-dried for 24 h.

### 2.3. FTIR Analyses

Fourier transform infrared (FTIR) spectroscopy (spectrum GX, Perkin Elmer, Waltham, MA, USA) was used to analyze the changes in functional groups of the WPAN with different surface modification steps. The WPAN, HWPAN, and PWPAN samples were prepared as KBr pellets and analyzed to verify the modification of the surface of the WPAN by hydrolysis and PEI coating in the frequency range 4000–400 cm^−1^. 

### 2.4. Batch Sorption Experiments

A Pt(IV) stock solution (1000 mg/L) was prepared by dissolving a weighed amount of H_2_PtCl_6_·5.5H_2_O in a 0.1 M HCl solution. Working solutions were prepared by a further dilution of the stock solution with 0.1 M HCl and used in the sorption experiments.

One-point sorption experiments was run by placing 0.03 g (dry weight) of sorbent into a 50 mL falcon tube and adding 30 mL of Pt solution (500 mg/L). The tubes were shaken at 160 rpm for 24 h at 25 °C in an incubator (HB-201MS-2R, Hanbaek Co., Bucheon, Korea). Isotherm sorption experiments were conducted under the same conditions, but with Pt(IV) solution concentration ranging from ~20–1000 mg/L. For the kinetic sorption experiment, a dry weight sorbent (0.1 g) was kept in a 250 mL beaker, followed by the addition of 100 mL of Pt solution (500 mg/L). Samples were taken at appropriate intervals for 7 h. 

The sorption uptake after each experiment was calculated from the mass balance—Equation (1). The initial and final Pt(IV) solutions after the sorption were diluted with distilled water, and the concentration of Pt(IV) was determined using ICP-AES (ICP-7500, Shimadzu, Kyoto, Japan).

(1) $$q\;=\;\frac{(C_{i}-C_{f})V}{M}$$

where *q* is the Pt(IV) uptake (mg/g); *C_i_* and *C_f_* are the initial and after-sorption concentrations (mg/L) of Pt(IV), respectively; *V* is the sorption working volume (L); *M* is mass (g) of the sorbent. 

Isotherm and kinetic data had standard errors less than 5%, which was confirmed by separate independent experimental runs.

### 2.5. Column Experiments

A column sorption experiment was performed using a 0.7854 mL sorbent-filled chromatography column (006SCC-10-10-FF, Omnifit, Danbury, CT, USA). A tube was connected to both ends of the column, and a peristaltic pump was used to pump the solution upwards through the column at a flow rate of 3 mL/min. Samples were taken at predetermined times over a period of 7 h.

## 3. Results and Discussion

### 3.1. Characterization of the PEI-Coated Waste PAN Textile

**Figure 1:**
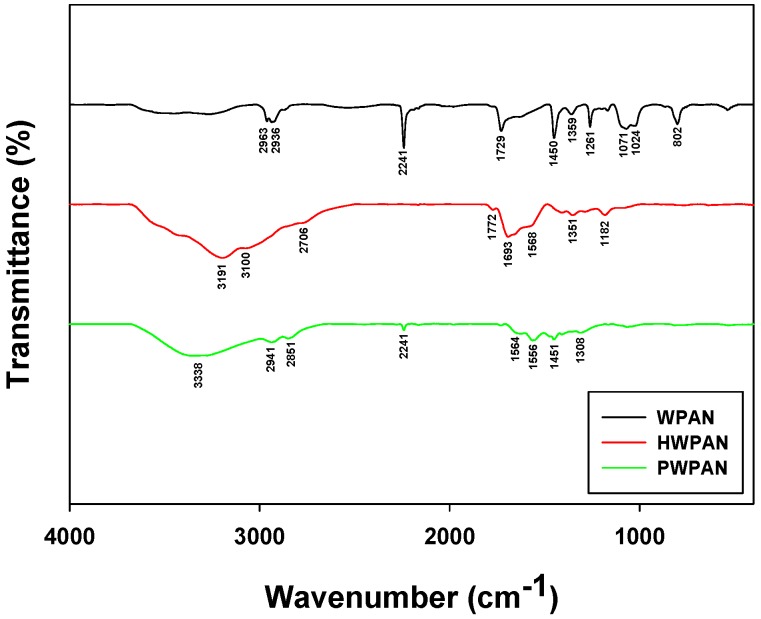
FTIR spectra of the WPAN, the HWPAN, and the PWPAN.

FTIR analyses were performed for WPAN and its modified forms (i.e., HWPAN and PWPAN) to characterize the surface modification and the presence of new functional groups. Based on a preliminary sorption test, the HWPAN and the PWPAN with a hydrolysis time of 120 min demonstrated the highest Pt(IV) uptake and hence were selected for the FTIR analyses. As shown in Figure 1, the FTIR spectrum of the WPAN showed the presence of a C≡N group and a CH_2_ bending symmetric vibration at 2241 and 1450 cm^−1^ [9]. After the hydrolysis of the WPAN, the C≡N groups disappeared, and CONH and COOH groups appeared to yield the HWPAN. The absorption bands for NH/OH, C=O, and NH in the FTIR spectrum of the WPAN appeared as combined stretching bands in the range 3600–2700 cm^−1^, 1693 cm^−1^, and 1565 cm^−1^, respectively [10]. The PEI used for coating was a branched type containing primary, secondary, and tertiary amine groups, and the peaks for these groups in the FTIR spectrum of the PWPAN were observed at the respective wavenumbers of 2851 cm^−1^, 2941 cm^−1^, and 3338 cm^−1^ [5].

**Figure 2:**
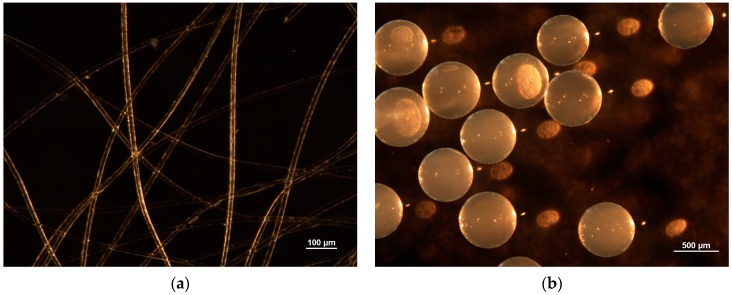
Microscopic images of (**a**) the PWPAN and (**b**) the Lewatit MonoPlus M500.

The surface morphologies and diameters of M500 and the PWPAN were observed using the stereoscopic microscope. The average diameter of the M500 (641.69 μm) was 43.5 times longer than that of the PWPAN (14.74 μm), as shown in Figure 2. In general, sorption occurs both on the surface and inside the ion-exchange resin. However, the sorption only plays on the surface of the PWPAN, because the ionic polymer was only coated on the outside of the fiber. Therefore, the diffusion distance of the PWPAN was estimated to be shorter than that of the M500. Because of the short diffusion distance, the kinetic of the PWPAN is faster than that of the M500. Furthermore, a high uptake of surface sorption needs a large surface area. The surface area of the PWPAN was 271.51 mm^2^/mm^3^, which was 29 times greater than that of the M500.

**Figure 3:**
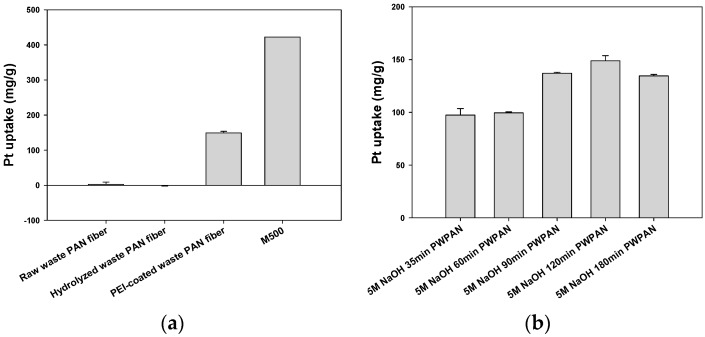
(**a**) Sorption effect of hydrolysis and PEI coating; (**b**) Sorption effect of the PWPAN hydrolysis time.

For comparing sorption abilities of the sorbents and investigating the effects of the surface modifications, Pt sorption experiments of the M500, the WPAN, the HWPAN, and the PWPAN were performed at an initial concentration of 500 mg/L (Figure 3a). The WPAN, the HWPAN, and the PWPAN showed a lower Pt(IV) uptake than the M500. It was also noted that the surface modification by the PEI coating (PWPAN) led to highly enhanced sorption compared to the WPAN and the HWPAN. As shown in Figure 3a, the uptakes of the WPAN and the HWPAN were almost close to zero, whereas the uptake of the PWPAN significantly increased to 148.80 mg/g. These results indicate that PEI was found to be effective for Pt(IV) recovery. Figure 3b also shows that the hydrolysis time of the PWPAN has a significant effect on Pt(IV) sorption. The Pt(IV) uptake increased from 97.46 to 148.80 mg/g with increasing hydrolysis time from 35 to 120 min. However, the uptake decreased (134.59 mg/g), with a further increase in time to 180 min. 

Sorption capacity of the WPAN and the HWPAN for Pt(IV), whose main species was [PtCl_6_]^2−^ in a 0.1 M HCl solution [11], was not observed, because their functional groups are mainly comprised of negative charges, e.g., cyanide and carboxyl, respectively. Meanwhile, the PWPAN with amine groups, which is ionizable under an acidic condition, can provide binding sites for the absorption of negatively charged Pt(IV) ions via electrostatic interactions. 

Figure 3b shows that the hydrolysis time affects the PEI coating reaction. The reaction mechanism involves the interaction of –NH_3_^+^ with –COO^−^. From 35 to 120 min, the attached PEI amount increased because of the increased carboxylic groups. Hence, the sorption capacity of Pt(IV) was improved. However, the backbone of the WPAN may be decomposed, dissolving carboxylic groups in the NaOH solution when the time exceeded 120 min. Therefore, a 120 min hydrolysis time for the PWPAN was selected for the subsequent isotherm, kinetics, and column experiments.

In general, the immobilization methods of the powder form of the biosorbent require a polymer solution. To dissolve these polymers, suitable organic solvents such as *N,N*-dimethylformamide, dimethyl sulfoxide, and ionic liquid are required [5,12,13]. However, most organic solvents are toxic. In contrast, the modification method of the PWPAN using a fibrous material was in the alkaline aqueous solution without using organic solvents. On the other hand, polyester can be produced from the carboxyl groups of a hydrolyzed reaction [14]. Therefore, this method provides a possible way to design a similar sorbent using different types of polyester textiles that are used in various fields.

### 3.2. Sorption Isotherm

**Figure 4:**
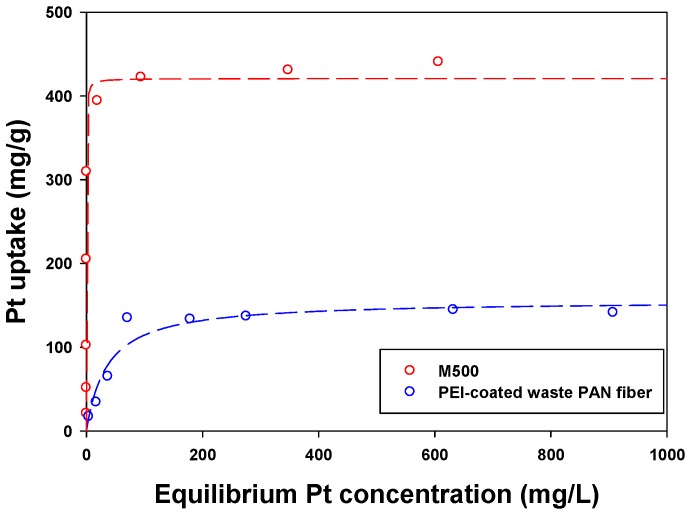
Sorption isotherms for the PWPAN and the Lewatit MonoPlus M500.

The sorption isotherm is one of the basic experiments for the evaluation of sorbent performance. It can obtain important parameters for maximum uptake and sorption affinity. Figure 4 shows the isotherm results of the PWPAN and the M500. At a low equilibrium Pt(IV) concentration, the uptake rate of Pt(IV) by the PWPAN was lower than that of the M500. At a high equilibrium Pt(IV) concentration, the uptake of the PWPAN was also lower than that of the M500. The Langmuir model was chosen to fit the isotherm data, and the equation is as follows:

(2) $$q_{e}=q_{m}bC_{e}/\left(1+bC_{e}\right)$$

where *q_e_* is the amount of the Pt(IV) adsorbed (mg/g), *q_m_* is the maximum uptake (mg/g), *b* is a sorption equilibrium constant (dm^3^/mg) related to the sorption affinity, and *C_e_* is the equilibrium concentration (mg/dm^3^).

The Langmuir model parameters are listed in Table 2. According to the Langmuir model, the maximum Pt(IV) uptake of the PWPAN and the M500 were 155.75 mg/g and 420.76 mg/g, respectively. Furthermore, the affinity constants b for the PWPAN and the M500 were 0.0278 L/mg and 5.4522 L/mg, respectively.

### 3.3. Sorption Kinetics

**Figure 5:**
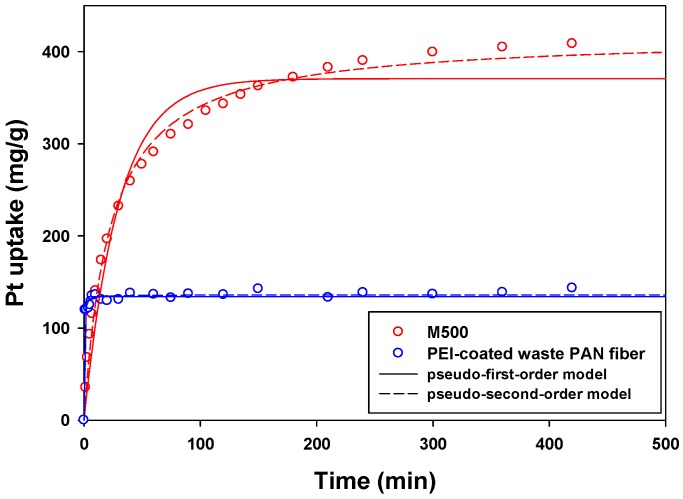
Sorption kinetics for the PWPAN and the Lewatit MonoPlus M500.

Sorption kinetic experiments were conducted to obtain the information of sorption rate, and it was especially important to check before applying the sorbent in the column systems. Figure 5 shows the adsorption kinetics of the PWPAN and the M500, fitted by pseudo-first-order and pseudo-second-order kinetic models (Table 2).

(3) $$q_{t}=q_{e}\left(1-\exp\left(-k_{1}t\right)\right)$$

(4) $$q_{t}=\frac{q_{e}^{2}k_{2}t}{1+q_{e}k_{2}t}$$

where *q_t_* is the uptake of Pt(IV) (mg/g) at time, *q_e_* is the uptake of Pt(IV) (mg/g) at equilibrium, *t* is the time (min), *k*_1_ is the pseudo-first-order rate constant (L/min), and *k*_2_ is the pseudo-second-order rate constant (g/mg/min). 

The R^2^ values of the pseudo-second-order kinetic model were higher than those of the pseudo-first-order kinetic model for both the sorbents. Therefore, the pseudo-second-order kinetic model was more appropriate in this kinetic study. Figure 5 shows that the Pt(IV) uptake of the PWPAN was 119.67 mg/g at 1 min, which almost reached the equilibrium uptake. The sorption rate constant (*k*_2_) of the PWPAN was 370 times higher than that of the M500. Compared to the previous study [5], the k_2_ value of PEI-PSBF (0.0003 g/mg/min) was much lower than that of the PWPAN (0.0370 g/mg/min) in this study, indicating that the PWPAN has a very fast sorption performance for Pt(IV). The fast kinetics of the PWPAN was an advantage for use with a low residence time in the column system, because the low residence time can decrease the sorbent packing volume or increase the flow rate. 

### 3.4. Column Experiments

At the industrial column design, the residence time is one of the major factors because it affects the packing volume (scale) and volumetric flow rate. Residence time reported in previous studies is generally long ~3.81 to 25 min [15,16,17,18]. In this study, an effective column process with a low residence time of 0.2618 min was developed using the fast kinetic PWPAN. The residence time was calculated by the following equation:

(5) $$\mathrm{Residence}\;\mathrm{time}\;=\;\frac{\mathrm{packing}\;\mathrm{volume}\;\mathrm{of}\;\mathrm{the}\;\mathrm{sorbent}}{\;\mathrm{volumetric}\;\mathrm{flow}\;\mathrm{rate}\;\mathrm{of}\;\mathrm{the}\;\mathrm{metal}\;\mathrm{solution}}$$

and bed volume (B.V.) was another major factor, which was calculated by the following equation:

(6) $$\mathrm{Bed}\;\mathrm{volume}\;=\;\frac{\mathrm{treated}\;\mathrm{solution}\;\mathrm{volume}\;}{\mathrm{packing}\;\mathrm{volume}\;\mathrm{of}\;\mathrm{the}\;\mathrm{sorbent}}.$$

**Figure 6:**
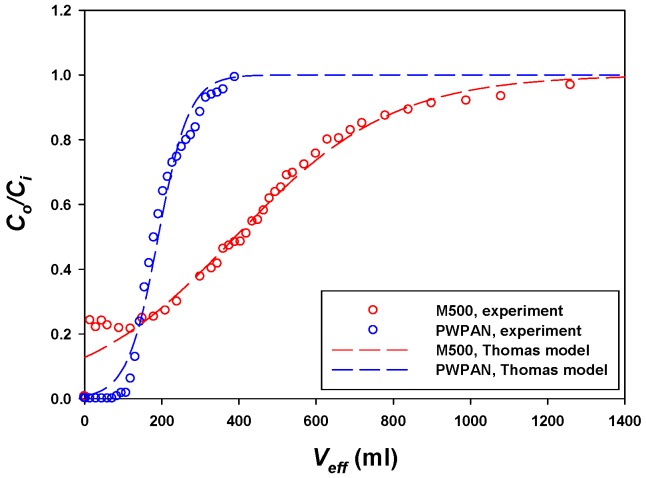
Sorption in column for the PWPAN and the Lewatit MonoPlus M500.

As a result, the breakthrough curves are shown in Figure 6. The criteria of the breakthrough point were evaluated on 1% of the inlet metal concentration (2.5 ppm). A typical Thomas model was employed to fit the column data, which can obtain a constant rate (*k_TH_*, mL/min/mg) and a maximum solid-phase concentration of the solute (*q*_0_, mg/g) [19]. The Thomas model is as follows:

(7) $$\frac{C_{0}}{C_{i}}=\frac{1}{1+\exp\left(\frac{k_{TH}}{Q}\left(q_{0}M-C_{i}V_{eff}\right)\right)}$$

where *C*_0_ is the Pt(IV) concentration (mg/mL) of the outlet, *C_i_* is the Pt concentration (mg/mL) of inlet, *Q* is the flow rate (mL/min) of the Pt(IV) solution, *M* is the mass (g) of the sorbent, and *V_eff_* is the effluent volume (mL). 

The Pt(IV) concentration of the outlet was >50 mg/L after 5 min using the commercial M500 resin, whereas the outlet concentration was <5 mg/L for 36 min (W.V. 137.5) using the PWPAN, indicating that 137.5 mL of 250 mg/L Pt(IV) could be recovered using 1 mL of the PWPAN. The Thomas model fit both the column data of the PWPAN and the M500 with an R^2^ > 0.97 (Table 2). Although the *q*_0_ value of the M500 (354.99 mg/g) was greater than that of the PWPAN (119.70 mg/g), the *k_TH_* value of the PWPAN (0.2925 mL/min/mg) was much higher than that of the M500 (0.0573 mL/min/mg). These results were in accordance with the isotherm and kinetic evaluations.

Through a comparison of Pt sorption capacities of the sorbents (Table 3), it is shown that the newly developed PWPAN has a higher Pt uptake than the activated carbon [20], commercial anion exchange resin (Diaion WA21J) [21], graphene oxide [22], and some artificially modified sorbents e.g., thiourea-modified chitosan [23], biopolymer modified activated carbon [20], polysulfone-based fibers [5], glycine-modified crosslinked chitosan resin [24], and PEI-modified *E. coli* [25]. Meanwhile, the sorption capacity of the PWPAN was lower than those of the M500, the ethylenediamine-modified magnetic chitosan nanoparticles [26], and some polymer-immobilized sorbents, e.g., polymer-immobilized polysulfone-based fibers [5], poly(allylamine hydrochloride)-modified *E. coli* [27], and poly(allylamine hydrochloride)-modified *E. coli*/chitosan fiber [28]. Here, the M500 has the highest Pt sorption ability, which is around 2.7 times higher than that of the PWPAN. Nevertheless, the sorption rate of the PWPAN was much faster than that of the M500. For this reason, the PWPAN shows superior sorption performance to the M500 when applying both sorbents in a column system at a low residence time.

## 4. Conclusions

The PWPAN was successfully developed using a simple surface modification method for Pt(IV) sorption. The hydrolysis time of 120 min was optimum for the preparation of the PWPAN. The maximum uptake of the PWPAN was 155.75 mg/g using the Langmuir model. The *k*_2_ value of the PWPAN was 0.0370 g/mg/min, which was 370 times higher than that of the M500 (0.0001 g/mg/min). The results of the column experiments further confirmed that the PWPAN could be applied in a column system at a low residence time (0.33 min) because of its fast kinetic property. Therefore, the PWPAN can be considered an alternative and effective sorbent for the recovery of Pt(IV) from aqueous solutions. From an economical or environmental perspective, the present study supports the development of low-cost and environmentally benign sorbents compared to activated carbons and other commercial sorbents, since the PWPAN could be developed using waste textiles and with low energy consumption.

## Figures and Tables

**Table 1:** Specification of the Lewatit MonoPlus M500.

Functional Group	Matrix	Structure	Mean Bead Size	Total Capacity
quaternary amine	crosslinked polystyrene	gel type	0.64 ± 0.05	1.3 eq/L

**Table 2:** The parameters determined by Langmuir isotherm, pseudo-first-order, pseudo-second-order, and Thomas models.

Sorbent	Langmuir Isotherm			Pseudo-First-Order			Pseudo-Second-Order			Thomas Model		
	$q_{m}$ (mg/g)	b (L/mg)	R^2^	$q_{1}$ (mg/g)	$k_{1}\;$ (L/min)	R^2^	$q_{2}$ (mg/g)	$k_{2}$ (g/mg/min)	R^2^	$k_{TH}$ (mL·min/mg)	$q_{0}\;$ (mg/g)	R^2^
M500	420.76	5.4522	0.81	370.62	0.0335	0.98	417.25	0.0001	0.99	0.0573	354.99	0.97
PWPAN	155.75	0.0278	0.92	133.97	2.0301	0.98	135.95	0.0370	0.99	0.2925	119.70	0.98

**Table 3:** Comparison of Pt sorption capacities of sorbents.

Sorbent	*q*_max_ (mg/g)	Reference
Activated carbon	45.5	[20]
Bio-polymer modified activated carbon	52.6	[20]
Polysulfone-based fibers	45.1	[5]
Polymer-immobilized polysulfone-based fibers	296.2	[5]
Poly(allylamine hydrochloride)-modified *E. coli*	348.8	[27]
Glycine-modified crosslinked chitosan resin	122.5	[24]
Thiourea-modified chitosan	129.9	[23]
Ethylenediamine-modified magnetic chitosan nanoparticles	171	[26]
Graphene Oxide	71.4	[22]
Diaion WA21J	5.69	[21]
Poly(allylamine hydrochloride)-modified *E. coli*/chitosan fiber	263.8	[28]
PEI-modified *E. coli*	108.8	[25]
PWPAN	155.75	Present study
M500	420.76	Present study
